# Clash of the -itises: An Unexpected Case of Sigmoid Colon Vasculitis

**DOI:** 10.14309/crj.0000000000000927

**Published:** 2022-12-26

**Authors:** Carolina Vigna, Stephen H. Wang, Ana Sofia Ore, Grant Eickel, Evangelos Messaris

**Affiliations:** 1 Division of Colorectal Surgery, Beth Israel Deaconess Medical Center, Harvard Medical School, Boston, MA

## Abstract

Vasculitis is an inflammatory process of the blood vessels, characterized by leukocyte infiltration in the vessel wall and reactive damage to the mural structures. They have a wide clinical spectrum and can present in a localized or systemic manner. Colonic involvement primarily manifests as abdominal pain and rectal bleeding. Less commonly, it can be associated with colonic perforation or anastomotic leakage after colorectal surgery. We report a case of a 42-year-old man with a history of HIV and proctocolitis who presented with an unexpected vasculitis of the sigmoid colon.

## INTRODUCTION

Vasculitides have a wide clinical spectrum and can present in a localized or systemic manner. Colonic involvement can be secondary to a small or medium-vessel systemic vasculitis, infection, connective tissue disorders, medication reaction, and less commonly an isolated single-organ vasculitis.^1,2^ We report a case of sigmoid colon vasculitis in a patient with proctocolitis and HIV, who developed an anastomotic leak after a sigmoid colectomy. Sigmoid colon vasculitis in patients on highly active antiretroviral therapy (HAART) and undetectable viral load is extremely rare and should be considered as a differential diagnosis before surgical intervention.

## CASE REPORT

A 42-year-old man with a history of HIV on HAART with undetectable viral load, anal condylomas, intersphincteric fistulas (status post condyloma excision and fistulectomies), perirectal abscesses, and ischemic proctitis (6 years ago) presented with 1 day of severe rectal pain associated with subjective fevers, nausea, several episodes of bloody and brown emesis, diarrhea with bright red blood mixed with clots, and fecal urge incontinence. The patient engages in receptive anal intercourse and was taking HIV and herpes simplex prophylactic medications.

Physical examination was notable for a fever of 38.9°C, distension and tenderness of the lower abdomen, and poor rectal sphincter tone. Significant laboratory findings included leukocytosis (WBC 29,000/mm^3^) and elevated lactate (2.5 mmol/L).

A computed tomography scan showed changes suggestive of severe proctocolitis. Blood, stool, and rectal swab cultures were obtained to evaluate for infectious etiologies; empiric antibiotic therapy was started.

**Figure 1.**
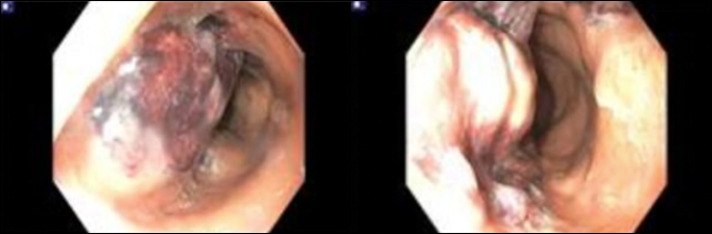
Sigmoidoscopy showing a submucosal circumferential mass in the proximal rectum/distal sigmoid colon.

**Figure 2.**
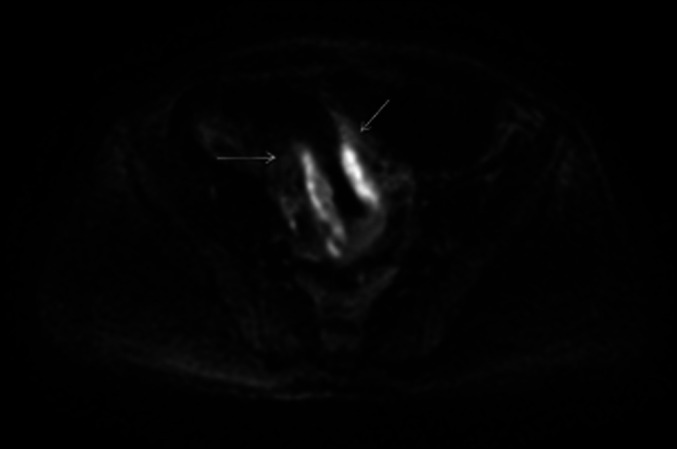
Pelvic MRI demonstrating a 10 cm segment of circumferential intramural abscess containing hemorrhage near the rectosigmoid junction, corresponding to abnormality seen on sigmoidoscopy.

Subsequently, he underwent sigmoidoscopy demonstrating a submucosal, circumferential, nonbleeding 5 cm mass 15–20 cm from the anal verge, suggestive of an abscess (Figure 1). A pelvic MRI revealed a 10 cm segment of circumferential intramural abscess near the rectosigmoid junction, corresponding to the lesion seen on sigmoidoscopy (Figure 2).

Stool studies and infectious serologies were negative. The patient improved clinically and remained afebrile, and his WBC count normalized. Etiology of the proctocolitis and intramural abscess were attributed to diverticulitis. He was discharged home after 1 week of hospitalization; surgical treatment was deferred to allow for abscess resolution.

Six weeks later, he underwent a laparoscopic sigmoid colectomy with primary end-to-end stapled anastomosis. On postoperative day 3, he developed sudden-onset abdominal pain, ileus, and increasing white blood cell count and C-reactive protein; anastomotic leak was suspected. Computed tomography with rectal contrast did not demonstrate leaks; however, inflammatory changes in the colon suggested segmental colitis.

The patient was taken to surgery for abdominal washout and creation of a diverting loop colostomy. Exploration of the peritoneal cavity demonstrated ascites and several adhesions, but no stool or purulent fluid was identified. Pelvic fluid collections were drained. Rigid proctoscopy was performed under water without evidence of air leak, suggestive of a contained anastomotic leak. The patient tolerated the procedure well and was discharged on postoperative day 10.

**Figure 3.**
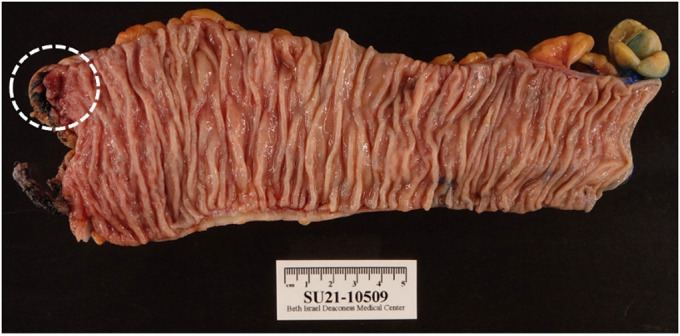
Gross specimen of the sigmoid colon showing focal erythema.

**Figure 4.**
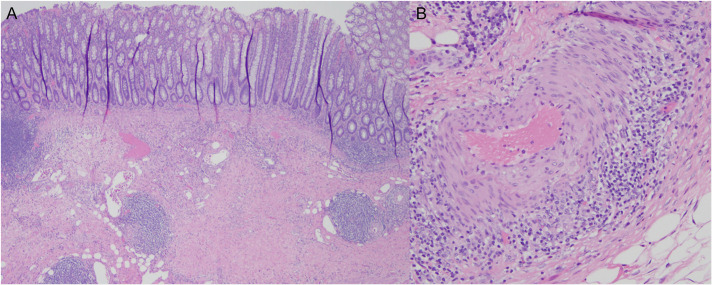
(A) Perivascular lymphocytic inflammation; (B) chronic vasculitis (predominantly lymphocytic).

Pathology evaluation of the sigmoid colon demonstrated patchy mild active colitis with focal perivascular lymphocytic inflammation and chronic vasculitis, involving the intramural and mesenteric arteries. No evidence of diverticular disease, granulomas, or viral cytopathic changes were identified (Figures 3 and 4).

Given that the pathology results demonstrated sigmoid colon vasculitis, rheumatology evaluation was performed. He was found to have bilateral numbness in the L4 dermatome distribution. Serology and electromyography were requested. Perinuclear anti-neutrophil cytoplasmic antibodies (p-ANCA) titers were elevated. He had normal serum protein electrophoresis and complement and negative anti-Ro and anti-LA, double stranded DNA, and anti-proteinase 3 anti-neutrophil cytoplasmic antibodies (PR3-ANCA) titers. Creatinine and urine analysis were within normal limits, so no kidney involvement was suspected. The patient most likely had isolated vasculitis, additionally supported by the fact that he remained asymptomatic after surgery. No medical therapy was initiated at the time; the patient will follow up in 6 months with rheumatology.

## DISCUSSION

Vasculitis is an inflammatory process of the blood vessels, characterized by leukocyte infiltration in the vessel wall, reactive damage to the mural structures, and alteration of blood flow to the affected organ. Within the gastrointestinal tract, vasculitis may cause ulceration, hemorrhage, ileus, mesenteric ischemia, and visceral perforation.^3^

Vasculitis is classified as primary or secondary. Primary vasculitides are divided into small, medium, and large-vessel based on size of the affected vessels. Secondary vasculitides are caused by infectious diseases (eg, HIV), connective tissue diseases, drugs, or malignancies.^2–4^ Single-organ vasculitis isolated to the gastrointestinal tract has been reported in the esophagus, stomach, gallbladder, pancreas, small intestine, and colon.^5^

Symptoms of vasculitis affecting the small bowel and colon are usually secondary to ischemia and subsequent infarction; they include abdominal pain and rectal bleeding, diarrhea, nausea, and vomiting.^6^ Less commonly, colonic perforation can occur, which may cause anastomotic leakage after colon resection.^7,8^

HIV-associated vasculitis in the post-HAART era is extremely rare, with a prevalence of approximately 1%. It can be secondary to direct vascular insult or immunologically mediated and most frequently occurs in severe immunocompromised patients (CD4 count <200 cells/mL), associated with opportunistic infections (including hepatitis, HTLV-1, CMV, tuberculosis, parvovirus, syphilis) or in the absence of identifiable causes.^9–11^ In this case, the pathology reported a predominantly lymphocytic vasculitis of the mesenteric and intramural arteries, which are middle-sized vessels. The vascular inflammation raises the possibility of systemic vasculitis such as idiopathic polyarteritis nodosa (PAN), a middle-sized vessel disease. The vasculitis may also be secondary to an infectious process. The fact that he had no systemic symptoms of inflammation and was asymptomatic for 6 weeks between the antibiotic therapy and the surgical intervention decreases the likelihood of idiopathic PAN.

PAN-like vasculitis in patients with HIV can present at any stage, without the classic waxing and waning symptomatology of PAN, and with peripheral neuropathy as a common presenting symptom.^12^ Furthermore, the immunocompromised state of patients with HIV makes them prone to opportunistic infections, which may cause a vasculitis.^10^ The fact that the patient has sensory deficit on bilateral legs after L4 dermatome distribution and is p-ANCA-positive again raises the possibility of a systemic vasculitis; nevertheless, positive p-ANCA and elevated MPO titers have been identified in HIV patients with associated infections.

Although the usual culprit of this clinical presentation is diverticulitis, one must not forget that other causes of inflammation can also lead to proctocolitis and abscess formation. Anastomotic leaks in the setting of inflammation, perforation, and abscess formation are not uncommon, with an incidence of 2%–7%.^13,14^ There is a paucity of data evaluating the incidence of anastomotic leaks in the setting of colonic vasculitis, an area that warrants investigation. If a diagnosis of systemic vasculitis was present before surgery, the question rises whether a temporary loop ileostomy would have been indicated. We present no evidence of systemic vasculitis found after thorough workup, but it should always be a differential in an unusual presentation of vasculitis such as a first event occurring in the bowel.

## DISCLOSURES

Author contributions: C. Vigna, SH Wang, and G. Eickel wrote the manuscript. AS Ore and E. Messaris revised the manuscript for intellectual content. All authors approved the final manuscript. E. Messaris is the article guarantor.

Financial disclosure: None to report.

Informed consent was obtained for this case report.
